# Prediction of Systemic Lupus Erythematosus Exacerbation in Patients with Clinical and Subclinical Musculoskeletal Inflammation

**DOI:** 10.3390/jcm14197063

**Published:** 2025-10-07

**Authors:** Rifat Medjedovic, Milan Bogojevic, Milica Markovic, Ivan Soldatovic

**Affiliations:** 1 Department of Rheumatology, Clinical Centre of Montenegro, 81000 Podgorica, Montenegro; milanbogojevic89@gmail.com (M.B.); minamarkovic@hotmail.com (M.M.); 2 Faculty of Medicine, University of Belgrade, 11000 Belgrade, Serbia

**Keywords:** systemic lupus erythematosus, ultrasound, musculoskeletal manifestations, subclinical inflammation

## Abstract

**Background/Objectives**: Systemic lupus erythematosus (SLE) is an autoimmune disease affecting multiple organ systems, characterized by remissions and relapses. Musculoskeletal involvement occurs in up to 95% of patients and may present as the initial symptom in 50%. Such involvement is often subclinical, without obvious joint or tendon inflammation. Musculoskeletal ultrasound (US) has proven valuable for detecting pathological changes in joints and periarticular structures, including in SLE patients, and early detection, particularly in subclinical stages, supports optimal therapy, monitoring, and improved prognosis. This study aimed to determine the frequency of new clinical manifestations in patients with previously confirmed clinical and subclinical musculoskeletal inflammation after 2 and 5 years, and to evaluate associations with sex, age, BMI, smoking status, ESR, CRP, SLEDAI-2K, complement components C3 and C4, anti-dsDNA antibodies concentrations, and prior treatment. **Methods**: The study included 34 SLE patients with clinical and 22 with subclinical musculoskeletal inflammation, confirmed at baseline by history, examination, and US. Follow-up at 2 and 5 years recorded new clinical manifestations. Correlations with patient characteristics were assessed to identify predictors. **Results**: New clinical manifestations occurred in 34% of patients at 2 years and 48% at 5 years, most commonly cutaneous, musculoskeletal, and hematological. Summary analysis identified female sex, lower BMI, and lower baseline SLEDAI-2K scores as the strongest predictors. In the subclinical group, female sex, smoking, and lower SLEDAI-2K scores were predictive, while in the clinical group, female sex, lower SLEDAI-2K scores, lower ESR, and higher anti-ds DNA levels were associated with new manifestations. **Conclusions**: Female sex, lower BMI, and lower baseline SLEDAI-2K scores are key predictors of new clinical manifestations in SLE patients, highlighting the importance of early detection and individualized monitoring, particularly in patients with subclinical musculoskeletal inflammation.

## 1. Introduction

Systemic lupus erythematosus (SLE) is a chronic autoimmune disorder with the potential to involve virtually any organ system. Its clinical course is typically marked by alternating periods of remission and flare, presenting a wide array of manifestations that may range from relatively mild musculoskeletal or cutaneous symptoms to serious, life-threatening complications affecting the cardiovascular, renal, or central nervous systems [1,2]. Musculoskeletal manifestations are among the most frequent features of SLE, observed at some stage in nearly all patients, and in about half, they represent the first clinical sign of disease [3]. Inflammation of the joints and tendons in SLE may or may not be detectable by clinical examination (arthritis, tenosynovitis, tendinitis, enthesitis, etc.), and it is frequently observed that patients with arthralgia lack overt clinical signs of inflammation [4]. Musculoskeletal ultrasound (MSUS) has demonstrated substantial value in detecting pathological changes in articular and periarticular structures in inflammatory rheumatic diseases, particularly in rheumatoid arthritis (RA) and psoriatic arthritis (PsA) [5]. Over time, MSUS has also proven to be valuable in patients with SLE and their musculoskeletal manifestations, not only in detection but also in defining pathogenetic patterns of musculoskeletal involvement [6]. Although musculoskeletal complaints are very common in SLE, true clinical synovitis is observed in a smaller subset of patients. The relationship between clinically evident synovitis and markers of disease activity has been investigated extensively, yet results remain inconsistent. In contrast, only limited research has addressed subclinical synovitis and its potential link to disease activity measures, which complicates disease monitoring and therapeutic decision-making [7]. Indeed, existing literature has demonstrated a high frequency of articular and periarticular manifestations in patients with SLE (58–100%). Nevertheless, due to the considerable proportion of clinically undetectable inflammation, ultrasound (US)-confirmed joint and tendon inflammation is considered suitable for clinical studies, treat-to-target monitoring strategies, and accurate assessment of disease activity [8,9]. Thus, US-confirmed joint and tendon inflammation is regarded as highly relevant to disease activity evaluation, especially considering its impact on quality of life and functional impairment [10,11]. The Systemic Lupus Erythematosus Disease Activity Index (SLEDAI), the most widely applied scoring system for assessing disease activity in daily clinical practice, does not encompass all potentially affected organs and systems. Moreover, musculoskeletal manifestations can contribute a maximum of only four points, and only in cases of clinically detectable inflammation [12]. Musculoskeletal ultrasound provides an opportunity for more precise detection of inflammation; however, previous studies have demonstrated substantial variability in pathological US findings, primarily due to methodological differences and lack of standardization [8]. Ultimately, a comprehensive understanding of the pathogenesis of musculoskeletal manifestations in SLE, their classification, and above all, early detection, appropriate treatment, and treatment monitoring, are key to suppressing inflammation, preventing deformities and disability, and improving quality of life [13]. Despite the availability of multiple therapeutic options, patients often experience relapses and remain at risk of organ damage [14]. Reliable prediction of disease activity changes is therefore of great importance. However, currently available clinical and serological markers show limited predictive capacity. Given the heterogeneity of disease mechanisms and manifestations, proactive monitoring approaches are warranted, and imaging techniques such as ultrasound are expected to play an increasingly important role [15,16]. Given that musculoskeletal manifestations of SLE are associated with poorer long-term outcomes, their early detection—particularly in subclinical stages—facilitates appropriate therapeutic selection, monitoring, and improved prognosis. Although MSUS has not yet been incorporated as a standard imaging modality for assessing joint activity in SLE, it undoubtedly holds considerable importance in the detection of inflammation [17].

The objectives of our study were to determine the incidence of new clinical manifestations of SLE after 2 years and 5 years of follow-up in patients with subclinical joint and tendon inflammation (without musculoskeletal symptoms or signs, but with ultrasound-confirmed inflammation) and in patients with clinical joint and tendon inflammation (with musculoskeletal symptoms and signs, and ultrasound-confirmed inflammation). Moreover we assessed the association (correlation) between the occurrence of new clinical manifestations of SLE, both in patients with subclinical and clinical inflammation, and the following characteristics: sex, age, body mass index (BMI) (including correlation with average BMI, as well as with underweight, normal, and obese categories), smoking status (smoker vs. non-smoker), erythrocyte sedimentation rate (ESR), c-reactive protein (CRP), SLEDAI-2K score and SLEDAI-2K categories of mild, moderate, high, and very high disease activity, complement components C3 and C4, anti-dsDNA antibodies, glucocorticoid use (yes/no) and average dose of glucocorticoids, and use of immunosuppressive drugs (azathioprine [AZA], methotrexate [MTX], mycophenolate).

## 2. Materials and Methods

In the first part of the study [17], a cross-sectional analysis was conducted on 61 patients to determine the prevalence of pathological findings on ultrasound of joints and tendons in patients with SLE, including both asymptomatic and symptomatic individuals (with respect to musculoskeletal symptoms and signs). Patients were followed and treated at the Institute of Rheumatology in Belgrade. The study was carried out from December 2017 to December 2019, following approval by the Ethics Committee of the institution (No 29/1-41, date: 2 March 2017). As in the initial phase of the study, eligibility required fulfillment of the 1997 revised American College of Rheumatology (ACR) classification criteria for SLE. Patients with overlapping connective tissue diseases, malignancy, pregnancy, rheumatoid arthritis overlap, or active infection were excluded, given that the present investigation represents a longitudinal extension of the original cohort. In the second part of the study, the patients’ status was also assessed cross-sectionally during follow-up at 2 and 5 years to verify the occurrence of new clinical manifestations of SLE (musculoskeletal and/or other clinical manifestations).

In the first part, the patient assessment included taking medical history (subjective complaints such as joint pain and/or painful joint swelling), clinical examination of SLE patients, review of the patient’s previous medical history, completion of a questionnaire, including demographic data, clinical and laboratory indicators of disease activity, calculation of the SLEDAI-2K, modified in 2000, and ultrasound examination. The total SLEDAI score can range from 0 to 105. According to the SLEDAI score, disease activity is assessed as follows: 0—no activity, 1–5—mild disease activity, 6–10—moderate disease activity, 11–19—high disease activity, 20 or more—very high disease activity [18].

Ultrasound examinations were performed using an ESAOTE MyLab 70 X-Vision V10.02 ultrasound system (VG). Imaging modalities included gray scale (GS) and power Doppler (PD). The ultrasound probes used were LA 523 (frequency range 4–13 MHz for elbows and knees) and LA 435 (frequency range 6–18 MHz for wrists, hands, ankles, and feet).

Ultrasound abnormalities of the joints were assessed according to the OMERACT (Outcome Measures in Rheumatology) group criteria using gray scale (GS) and power Doppler (PD) modalities and included joint effusion (nominal scale—present/absent), synovial hypertrophy (GS definition and scoring), and synovitis with PD signal (PD scoring). Ultrasound abnormalities of the tendons, also according to OMERACT criteria (GS and PD), included tendon effusion (nominal scale—present/absent), tenosynovitis with PD signal (definition and PD scoring), and partial or complete tendon rupture (binary system—present/absent).

Joint and tendon ultrasound examinations were performed concurrently for all patients by two experienced physicians independently.

In the second part of the study, at 2 years (i.e., during 2021) and 5 years (i.e., during 2024), the patients’ medical history and documentation were reviewed (including outpatient rheumatology follow-up visits, any hospitalizations, and relevant laboratory and other investigations) to determine whether new clinical manifestations of SLE had occurred. This evaluation was conducted for both asymptomatic and symptomatic patients (with respect to musculoskeletal symptoms and signs).

Results are presented as count (%), mean ± standard deviation or median (interquartile range), depending on data type and distribution. Groups were compared using parametric (*t*-test) and nonparametric (Chi-square, Mann–Whitney U test) tests. Logistic regressions were performed to evaluate the relationship between the dependent variable and independent variables. All *p*-values less than 0.05 were considered significant. All data were analyzed using SPSS 29.0 (IBM Corp. Released 2023. IBM SPSS Statistics for Windows, Version 29.0. Armonk, NY, USA: IBM Corp.) and R 4.1.0. (R Core Team (2017)). R: A language and environment for statistical computing. R Foundation for Statistical Computing, Vienna, Austria. URL https://www.R-project.org/, accessed on 18 August 2025.

## 3. Results

The results and conclusions of the first part of this cross-sectional study were published in July 2023 [17]. Based on subjective complaints (joint pain and/or painful swelling), objective findings (palpatory tenderness and/or joint swelling), and ultrasound (US) findings, patients were divided into four groups:

1. Patients with subjective complaints and/or objective findings who also had positive ultrasound findings (clinical inflammation);
2. Patients without subjective complaints or objective findings who had positive ultrasound findings (subclinical inflammation);
3. Patients without subjective complaints or objective findings who had negative ultrasound findings;
4. Patients with subjective complaints and/or objective findings who had negative ultrasound findings.

When patients were classified in this manner, out of a total of 61 patients,

- 34 patients had subjective complaints and/or objectively positive findings along with positive US findings (clinical inflammation);
- 22 patients had no subjective complaints or objective findings but had positive US findings (subclinical inflammation);
- Only 3 patients had neither subjective complaints nor objective findings and had negative US findings;
- 2 patients had subjective complaints or objective findings but negative US findings.

Both in the first part of this cross-sectional study and in the subsequent 2- and 5-year follow-up analyses, only patients with clinical inflammation (34 patients) and subclinical inflammation (22 patients) were included in the analysis, while the remaining two groups were excluded due to the small number of patients [17]. As previously reported, the highest prevalence of subclinical inflammation was observed in the wrist region (joints and extensor tendons) and the knees. Regarding the wrist joints, ultrasound-confirmed inflammation was higher in the group with clinically evident findings, but it was also notable in the group without subjective complaints and objective signs (32% effusion/synovial hypertrophy and 5% PD signal in patients without subjective complaints; 39% effusion/synovial hypertrophy and 4.9% PD signal in patients without objective findings). In the knees, a considerable proportion of patients without subjective complaints or objective musculoskeletal findings also demonstrated US evidence of inflammation (37.5% effusion/synovial hypertrophy and 8.3% PD signal in patients without subjective complaints; 39.7% effusion/synovial hypertrophy and 12.1% PD signal in patients without objective findings). For the extensor tendons of the wrist, a significant proportion of subclinical inflammation was observed (30.8% effusion/PD signal in patients without subjective complaints; 40.7% effusion/PD signal in patients without objective findings). The association between subclinical inflammation and SLEDAI-2K scores, complement components C3 and C4, anti-ds DNA antibodies concentrations, ESR, and CRP was evaluated, and no statistically significant differences were found when comparing patients with clinical versus subclinical inflammation [17].

The 2- and 5-year follow-up study demonstrated that among these two groups of patients (34 with clinical inflammation and 22 with subclinical inflammation, totaling 56 patients), new clinical manifestations of SLE occurred in 19 patients (34%) after 2 years of follow-up and in 27 patients (48%) after 5 years of follow-up.

At the 2-year follow-up, 10 patients developed cutaneous manifestations, 4 patients developed musculoskeletal manifestations, and 5 patients had hematological manifestations of the disease (with 3 patients exhibiting isolated hematological manifestations).

At the 5-year follow-up, 12 patients developed cutaneous manifestations, 7 patients developed new hematological manifestations (5 with isolated hematological manifestations, 2 with both hematological manifestations and arthritis), 7 patients developed new musculoskeletal manifestations, 1 patient presented with serositis, 1 patient with lupus nephritis, and 1 patient with neuro-lupus.

The study included 56 participants with clinical (n = 34) or subclinical (n = 22) forms of the disease, who were predominantly female (n = 48; 85.7%), with an average age of 47.0 ± 14.9 years, which ranged between a minimum of 21 years and maximum of 58 years. Differences between the two forms of the disease are presented in Table 1. The subclinical form was predominantly female compared with the clinical form, and had a higher percentage of smokers, but similar age, BMI, SE and CRP. Disease activity (SLEDAI) was almost identical, but C3 and C4 were higher in the subclinical form. In both the 2- and 5-year follow-ups, new symptoms were more prevalent in subclinical form, but no significance was obtained in any of the presented analyses in Table 1.

The predictors of the main outcomes at the 2- and 5-year follow-up for new symptoms, regardless of form, are presented in Table 2. Both outcomes were more prevalent in females and in those with a lower BMI. Participants with new onset had a lower SLEDAI in both outcomes. The therapy distribution revealed no differences in outcomes, especially due to the small number of participants in specific categories.

The predictors of the main outcomes a the 2- and 5-year follow-ups for new symptoms in patients with the clinical form at baseline are presented in Table 3. Female gender had more prevalent outcomes compared to males, but no differences regarding age and BMI were observed. Smokers had less prevalent outcomes compared with non-smokers. Participants with outcomes had a lower SLEDAI, as well as SE, but higher anti-ds DNK Ab.

The predictors of the main outcomes at the 2- and 5-year follow-ups for new symptoms in patients with the subclinical form at baseline are presented in Table 4. Female gender was dominant in this subgroup. No differences regarding age and BMI were observed, similar to the clinical form. Smokers had more prevalent outcomes compared with non-smokers, which is opposite to the clinical form. Participants with outcomes had lower SLEDAI, C3, C4, and anti-ds DNA antibodies.

Modeling of new symptom onset during the 2- and 5-year follow-ups is presented in Table 5. Modeling was performed in two steps. All predictors that had any *p*-values of less than 0.2 in previous analyses were included in the analysis. Using the backward method, eliminating variables one at a time was performed to assess variables with a *p*-value of less than 0.1. The final models are presented in table and revealed that female gender, BMI, and SLEDAI were the most important predictors. According to the model, the female gender was six times more likely to have new symptoms during follow-up, while participants with a higher BMI and SLEDAI were less likely to have new symptoms during follow-up.

## 4. Discussion

The results of the first part of this cross-sectional study [17] were largely consistent with the frequency distributions and associations described in earlier systematic reviews by Zayat et al. and Di Matteo et al. [8,19]. Of particular note was that 36% of patients in the studied cohort exhibited subclinical musculoskeletal inflammation. When compared with the aforementioned systematic reviews and the studies included therein, this proportion is very similar. Analysis using the Mann–Whitney U test did not reveal statistically significant differences between patients with clinical and subclinical inflammation regarding SLEDAI-2K scores, complement components C3 and C4, anti-ds DNA antibodies, ESR, or CRP. However, in the subclinical inflammation group, the values for C3, C4, anti-ds DNA antibodies, ESR, CRP, and SLEDAI scores were very close to those observed in patients with clinically evident inflammation confirmed by history, objective examination, and ultrasound (clinical inflammation). A high prevalence of subclinical musculoskeletal inflammation was also reported in the study by Ruano et al., with rates ranging from 60% to 70%, depending on the stage of synovitis [20].

In the longitudinal phase of the study, which included patients with clinical (34 patients) and subclinical (22 patients) musculoskeletal inflammation, the subclinical inflammation group showed a slightly higher proportion of women and smokers, as well as slightly higher values for complement components C3 and C4. At both the 2-year and 5-year follow-ups, new clinical manifestations were more frequently observed in patients with subclinical inflammation. Although these differences were not statistically significant, this finding may be interpreted in light of the fact that smoking is a well-established risk factor for the development of inflammatory rheumatic diseases, including SLE, and that female sex is also considered a risk factor [21]. The higher incidence of new clinical manifestations in patients with subclinical musculoskeletal inflammation may potentially be related to the absence of appropriately intensified immunosuppressive therapy [22]. The observed higher values for C3 and C4 in the subclinical inflammation group can be explained by the fact that decisions regarding immunosuppressive treatment are often based on the analysis of immunological disease activity in SLE. Therefore, in patients with subclinical and potentially unrecognized musculoskeletal inflammation, adequate immunosuppressive therapy is frequently lacking. Importantly, one possible explanation for the higher incidence of new clinical manifestations of SLE after 2 and 5 years in patients with subclinical inflammation is the inability to precisely detect inflammation at the subclinical stage, resulting in a lack of dose escalation of existing medications and/or the introduction of additional immunosuppressive agents. To date, the prognostic significance of subclinical musculoskeletal inflammation in SLE remains uncertain. Some studies have suggested a relationship between ultrasound-confirmed synovitis or tenosynovitis and disease activity scores such as SLEDAI-2K or the Health Assessment Questionnaire Disability Index (HAQ-DI). In studies by Abdel-Magied et al. [23], Ahmed Zayat et al. [8], and Torrente-Segarra et al. [7], such correlations were demonstrated; however, these studies did not assess the occurrence of new clinical manifestations. Conversely, no statistically significant correlation between ultrasound-confirmed musculoskeletal inflammation and SLEDAI-2K scores or HAQ-DI was reported in studies by Iagnocco et al. [24] and Delle Sedie et al. [25]. This indicates that the topic remains unresolved and warrants further investigation. Interestingly, Ruano et al. observed that asymptomatic SLE patients may demonstrate higher ultrasound-derived synovial hypertrophy indices than symptomatic individuals, suggesting that treatment approaches could differ substantially depending on whether subclinical findings are recognized [20]. Among both patients with clinical and subclinical inflammation at baseline, the occurrence of new clinical manifestations in both groups—after 2 and 5 years of follow-up—was more frequent in female patients and in those with lower BMI, as well as in patients who initially had lower SLEDAI-2K scores. Previous therapeutic interventions did not significantly influence the outcomes of new clinical manifestations at the 2- and 5-year follow-ups, particularly due to the small number of patients in individual categories.

In the group of patients with clinical inflammation at baseline, the occurrence of new clinical manifestations after both 2 and 5 years of follow-up was more frequent in women and slightly more frequent in those with lower baseline SLEDAI-2K scores, lower ESR, but higher levels of anti-ds DNA antibodies at study entry.

In the group of patients with subclinical inflammation at baseline, the occurrence of new clinical manifestations after 2 and 5 years of follow-up was also more frequent in women; however, unlike the clinical inflammation group, new manifestations were more common among smokers. Additionally, in this subclinical group, new clinical manifestations were more frequently observed in patients with lower baseline SLEDAI-2K scores.

A summary analysis of both patient groups revealed that the strongest predictors of new clinical manifestations in patients with SLE were female sex (six times more frequent, at the borderline of statistical significance), lower BMI (statistically significant), and lower baseline SLEDAI-2K scores (at the borderline of statistical significance). Regarding female sex as a predictor of future new SLE manifestations, one explanation is that SLE occurs nine times more frequently in women, and elevated estrogen levels, along with hormonal changes, may act as a trigger for disease exacerbation in females [26,27]. The finding that new clinical manifestations were more frequent in patients with a lower BMI after the 2- and 5-year follow-ups is somewhat paradoxical, as higher BMI, particularly in the obese range, is generally considered a risk factor for SLE development and associated with poorer prognosis, higher disease activity, and more frequent onset or worsening of lupus nephritis [28,29]. However, this observation may reflect limitations of the second part of the study, where BMI data at the 2- and 5-year follow-ups were not available. A possible explanation is related to the pharmacokinetics of medications used in SLE treatment; in patients with obesity, drugs may have a different pharmacokinetic profile with gradual release from adipose tissue where they are stored, often requiring higher doses. Additionally, more potent immunosuppressive therapy may have been administered to patients with obesity at baseline due to appropriate disease prognosis assessment. Similarly, the finding that patients with higher baseline SLEDAI-2K scores experienced new clinical manifestations less frequently during follow-up can be explained by the fact that patients with higher initial disease activity scores are generally treated with more potent immunosuppressive therapy from the outset, regardless of the type of SLE manifestation.

The main limitations of this study are primarily related to the relatively small sample size, which reflects the rarity of SLE, the cross-sectional design of the baseline study, and the fact that all patients were recruited from a single center. The 2- and 5-year follow-ups focused only on the occurrence of new clinical manifestations based on patient medical records and did not include repeated demographic, clinical, or ultrasound evaluations, which were available only at baseline. These constraints likely contributed to the absence of statistical significance in several analyses and may also explain the paradoxical observation that patients with a higher baseline BMI developed fewer new manifestations during follow-up. In addition, the number of patients with individual manifestations was limited, and particularly severe organ involvement was observed only in isolated cases (one patient with serositis, one with lupus nephritis, and one with CNS lupus). Due to this very low frequency, a separate statistical analysis of these outcomes was not feasible, and all new SLE manifestations were therefore analyzed collectively.

## 5. Conclusions

The conclusions of the first part of this study, published in July 2023 [17], demonstrated that the highest prevalence of subclinical inflammation in this group of SLE patients was observed in the wrist region (joints and extensor tendons) and the knees. Analysis of the association between subclinical inflammation and SLEDAI-2K scores, complement components C3 and C4, anti-dsDNA antibodies, ESR, and CRP did not reveal statistically significant differences between patients with clinical and subclinical inflammation.

In the second part of the study, encompassing the 2- and 5-year follow-ups of patients with SLE with baseline subclinical and clinical musculoskeletal inflammation, summary analysis of both patient groups identified the strongest predictors of new clinical manifestations as female sex (at the borderline of statistical significance), lower BMI (statistically significant), and lower SLEDAI-2K scores (at the borderline of statistical significance).

Specifically, in the subclinical inflammation group, the strongest predictors of new clinical manifestations were female sex, smoking status, and lower SLEDAI-2K scores (not statistically significant). In the clinical inflammation group, the strongest predictors were female sex, lower SLEDAI-2K scores, lower ESR, and higher levels of anti-dsDNA antibodies (not statistically significant).

## Figures and Tables

**Table 1:** Characteristics of participants with clinical and subclinical forms of the disease.

	Clin (n = 34)	Subclin (n = 22)	*p*-Value
Gender male	7 (20.6%)	1 (4.5%)	0.130 ^c^
Age	48.2 ± 16.4	45.2 ± 12.4	0.465 ^b^
BMI	24.8 ± 4.9	25.3 ± 6.3	0.732 ^b^
Smoking	11 (32.4%)	11 (50%)	0.187 ^a^
SE	16.0 (22.0)	16.5 (26.0)	0.518 ^d^
CRP	11.0 (27.0)	13.0 (23.9)	0.712 ^d^
SLEDAI	12 (15)	12 (13)	0.762 ^d^
Mild	4 (11.8%)	3 (13.6%)	0.951 ^d^
Moderate	10 (29.4%)	6 (27.3%)	
High	9 (26.5%)	6 (27.3%)	
Very high	11 (32.4%)	7 (31.8%)	
C3	1.6 (72.1)	9.0 (88.8)	0.847 ^d^
C4	0.2 (10.9)	10.0 (17.1)	0.185 ^d^
Anti-dsDNA ab	152.0 (529.7)	157.6 (537.4)	0.808 ^d^
GC	32 (94.1%)	21 (95.1%)	1.000 ^c^
Antimalarial	29 (85.3%)	19 (90.5%)	0.696 ^c^
MTX	5 (14.7%)	2 (9.1%)	0.692 ^c^
AZA	11 (32.4%)	6 (27.3%)	0.686 ^a^
Mycophenolate	0	1 (4.5%)	0.393 ^c^
2 years clin	10 (29.4%)	9 (40.9%)	0.375 ^a^
5 years clin	15 (44.1%)	12 (54.5%)	0.446 ^a^

^a^ Pearson Chi-square; ^b^ *t*-test; ^c^ Fisher’s exact test; ^d^ Mann–Whitney U test.

**Table 2:** New symptoms during 2- and 5-year follow-ups.

	2-Years FU		*p*-Value	5-Years FU		*p*-Value
	No (n = 37)	Yes (n = 19)		No (n = 29)	Yes (n = 27)	
Gender male						
Male	6 (75%)	2 (25%)	0.703 ^c^	6 (75%)	2 (25%)	0.254 ^c^
Female	31 (64.6%)	17 (35.4%)		23 (47.9%)	25 (52.1%)	
Age	46.6 ± 13.3	48.0 ± 18.0	0.737 ^b^	47.7 ± 13.3	46.4 ± 16.7	0.757 ^b^
BMI	25.7 ± 5.8	23.6 ± 4.5	0.181 ^b^	26.0 ± 6.0	23.9 ± 4.6	0.160 ^b^
Smoking						
No	23 (67.6%)	11 (32.4%)	0.757 ^a^	18 (52.9%)	16 (47.1%)	0.830 ^a^
Yes	14 (63.6%)	8 (36.4%)		11 (50%)	11 (50%)	
SE	16 (22)	16 (20)	0.903 ^d^	20 (20)	16 (20)	0.495 ^d^
CRP	12 (28.8)	10.75 (13.2)	0.501 ^d^	11.8 (28.8)	11 (17)	0.661 ^d^
SLEDAI	12 (14)	10 (12)	0.153 ^d^	14 (17)	11 (10)	0.181 ^d^
Mild	3 (42.9%)	4 (57.1%)	0.135 ^e^	3 (42.9%)	4 (57.1%)	0.200 ^e^
Moderate	10 (62.5%)	6 (37.5%)		7 (43.8%)	9 (56.3%)	
High	10 (66.7%)	5 (33.3%)		7 (46.7%)	8 (53.3%)	
Very high	14 (77.8%)	4 (22.2%)		12 (66.7%)	6 (33.3%)	
C3	9 (88.04)	1.03 (83.23)	0.299 ^d^	9 (77)	1.6 (91.23)	0.436 ^d^
C4	2 (13.83)	0.22 (10.88)	0.426 ^d^	2 (11.82)	0.28 (13.89)	0.682 ^d^
Anti-dsDNK ab ^f^	191.2 (481.6)	124 (574.2)	0.568 ^d^	156.5 (297)	152 (614)	0.555 ^d^
GC						
No	2 (66.7%)	1 (33.3%)	1.000 ^c^	2 (66.7%)	1 (33.3%)	1.000 ^c^
Yes	35 (66%)	18 (34%)		27 (50.9%)	26 (49.1%)	
Antimalarial						
No	3 (42.9%)	4 (57.1%)	0.219 ^c^	3 (42.9%)	4 (57.1%)	0.705 ^c^
Yes	33 (68.8%)	15 (31.3%)		25 (52.1%)	23 (47.9%)	
MTX						
No	33 (67.3%)	16 (32.7%)	0.679 ^c^	26 (53.1%)	23 (46.9%)	0.700 ^c^
Yes	4 (57.1%)	3 (42.9%)		3 (42.9%)	4 (57.1%)	
AZA						
No	25 (64.1%)	14 (35.9%)	0.637 ^a^	20 (51.3%)	19 (48.7%)	0.909 ^a^
Yes	12 (70.6%)	5 (29.4%)		9 (52.9%)	8 (47.1%)	
Mycophenolate						
No	36 (65.5%)	19 (34.5%)	1.000 ^c^	28 (50.9%)	27 (49.1%)	1.000 ^c^
Yes	1 (100%)	0 (0%)		1 (100%)	0 (0%)	

^a^ Pearson Chi-square; ^b^ *t*-test; ^c^ Fisher’s exact test; ^d^ Mann–Whitney U test; ^e^ Chi-square test for trend. Results are presented as count (%), mean ± standard deviation, or median (interquartile range), ^f^ antibodies

**Table 3:** New symptoms during 2- and 5-year follow-up in patients with clinical form at baseline.

	2-Years FU		*p*-Value	5-Years FU		*p*-Value
	No (n = 24)	Yes (n = 10)		No (n = 19)	Yes (n = 15)	
Gender male						
Male	6 (85.7%)	1 (14.3%)	0.644 ^c^	6 (85.7%)	1 (14.3%)	0.104 ^c^
Female	18 (66.7%)	9 (33.3%)		13 (48.1%)	14 (51.9%)	
Age	47.0 ± 14.9	51.1 ± 20.1	0.519 ^b^	48.5 ± 15.4	47.9 ± 18.1	0.926 ^a^
BMI	24.9 ± 5.3	24.6 ± 4.1	0.866 ^b^	25.0 ± 5.2	24.6 ± 4.8	0.789 ^a^
Smoking						
No	14 (60.9%)	9 (39.1%)	0.113 ^c^	11 (47.8%)	12 (52.2%)	0.271 ^c^
Yes	10 (90.9%)	1 (9.1%)		8 (72.7%)	3 (27.3%)	
SE	18.0 (22.0)	15.5 (11.0)	0.609 ^d^	22 (30)	14 (12)	0.110 ^d^
CRP	11.4 (27.0)	10.5 (5.5)	0.840 ^d^	11.0 (25.5)	10.7 (25.5)	0.884 ^d^
SLEDAI	13 (17)	11 (11)	0.325 ^d^	14 (20)	11 (10)	0.466 ^d^
Mild	2 (50%)	2 (50%)	0.206 ^e^	2 (50%)	2 (50%)	0.409 ^e^
Moderate	7 (70%)	3 (30%)		5 (50%)	5 (50%)	
High	5 (55.6%)	4 (44.4%)		4 (44.4%)	5 (55.6%)	
Very high	10 (90.9%)	1 (9.1%)		8 (72.7%)	3 (27.3%)	
C3	9.0 (72.1)	1.3 (91.2)	0.416 ^d^	1.0 (67.1)	8.0 (94.2)	1.000 ^d^
C4	1.1 (11.3)	0.2 (10.9)	0.508 ^d^	0.23 (7.83)	1.00 (13.91)	0.603 ^d^
Anti-dsDNK ab ^f^	112.0 (367.9)	262.0 (645.0)	0.584 ^d^	86.0 (322.4)	313.0 (707.7)	0.176 ^d^
GC						
No	2 (100%)	0 (0%)	1.000 ^c^	2 (100%)	0 (0%)	0.492 ^c^
Yes	22 (68.8%)	10 (31.3%)		17 (53.1%)	15 (46.9%)	
Antimalarial						
No	3 (60%)	2 (40%)	0.618 ^c^	3 (60%)	2 (40%)	1.000 ^c^
Yes	21 (72.4%)	8 (27.6%)		16 (55.2%)	13 (44.8%)	
MTX						
No	20 (69%)	9 (31%)	1.000 ^c^	16 (55.2%)	13 (44.8%)	1.000 ^c^
Yes	4 (80%)	1 (20%)		3 (60%)	2 (40%)	
AZA						
No	15 (65.2%)	8 (34.8%)	0.437 ^c^	12 (52.2%)	11 (47.8%)	0.715 ^c^
Yes	9 (81.8%)	2 (18.2%)		7 (63.6%)	4 (36.4%)	
Mycophenolate						
No	24 (70.6%)	10 (29.4%)	/	19 (55.9%)	15 (44.1%)	/
Yes	0 (0%)	0 (0%)		0 (0%)	0 (0%)	

^a^ Pearson Chi-square; ^b^ *t*-test; ^c^ Fisher’s exact test; ^d^ Mann–Whitney U test; ^e^ Chi-square test for trend. Results are presented as count (%), mean ± standard deviation, or median (interquartile range), ^f^ ab -antibodies.

**Table 4:** New symptoms during 2- and 5-year follow-ups in patients with the subclinical form at baseline.

	2-Years FU		*p*-Value	5-Years FU		*p*-Value
	No (n = 13)	Yes (n = 9)		No (n = 10)	Yes (n = 12)	
Gender male						
Male	0 (0%)	1 (100%)	0.409 ^c^	0 (0%)	1 (100%)	1.000 ^c^
Female	13 (61.9%)	8 (38.1%)		10 (47.6%)	11 (52.4%)	
Age	45.7 ± 10.2	44.6 ± 15.7	0.838 ^a^	46.1 ± 8.7	44.5 ± 15.2	0.761 ^a^
BMI	27.2 ± 6.6	22.6 ± 4.9	0.091 ^a^	27.9 ± 7.4	23.2 ± 4.5	0.100 ^a^
Smoking						
No	9 (81.8%)	2 (18.2%)	0.080 ^c^	7 (63.6%)	4 (36.4%)	0.087 ^a^
Yes	4 (36.4%)	7 (63.6%)		3 (27.3%)	8 (72.7%)	
SE	15 (20)	26 (20)	0.593 ^d^	14.5 (20.0)	21.5 (23.5)	0.391 ^d^
CRP	14.0 (30.0)	11 (13.2)	0.504 ^d^	16.0 (39.0)	11.5 (16.8)	0.373 ^d^
SLEDAI	12 (10)	9 (14)	0.403 ^d^	14.5 (15.0)	11.0 (10.5)	0.290 ^d^
Mild	1 (33.3%)	2 (66.7%)	0.547 ^e^	1 (33.3%)	2 (66.7%)	0.429 ^e^
Moderate	3 (50%)	3 (50%)		2 (33.3%)	4 (66.7%)	
High	5 (83.3%)	1 (16.7%)		3 (50%)	3 (50%)	
Very high	4 (57.1%)	3 (42.9%)		4 (57.1%)	3 (42.9%)	
C3	10 (90.88)	1 (83.1)	0.385 ^d^	11.0 (90.4)	0.9 (83.3)	0.166 ^d^
C4	11 (17.79)	0.28 (10.82)	0.442 ^d^	12.5 (18.7)	0.2 (13.4)	0.129 ^d^
Anti-dsDNK ab ^f^	326 (493.6)	83 (110)	0.124 ^d^	269.0 (478.0)	92.0 (554.1)	0.410 ^d^
GK						
No	0 (0%)	1 (100%)	0.409 ^c^	0 (0%)	1 (100%)	1.000 ^a^
Yes	13 (61.9%)	8 (38.1%)		10 (47.6%)	11 (52.4%)	
Antimalarial						
No	0 (0%)	2 (100%)	0.171 ^c^	0 (0%)	2 (100%)	0.486 ^c^
Yes	12 (63.2%)	7 (36.8%)		9 (47.4%)	10 (52.6%)	
MTX						
No	13 (65%)	7 (35%)	0.156 ^c^	10 (50%)	10 (50%)	0.481 ^c^
Yes	0 (0%)	2 (100%)		0 (0%)	2 (100%)	
AZA						
No	10 (62.5%)	6 (37.5%)	0.655 ^c^	8 (50%)	8 (50%)	0.646 ^c^
Yes	3 (50%)	3 (50%)		2 (33.3%)	4 (66.7%)	
Mycophenolate						
No	12 (57.1%)	9 (42.9%)	1.000 ^c^	9 (42.9%)	12 (57.1%)	0.455 ^c^
Yes	1 (100%)	0 (0%)		1 (100%)	0 (0%)	

^a^ Pearson Chi-square; ^c^ Fisher’s exact test; ^d^ Mann–Whitney U test; ^e^ Chi-square test for trend. Results are presented as count (%), mean ± standard deviation, or median (interquartile range), ^f^ antibodies.

**Table 5:** Logistic regression models with new symptoms as outcome.

	New Symptoms			
	2-Years FU	*p*-Value	5-Years FU	*p*-Value
	OR (95% CI)		OR (95% CI)	
Age	1.048 (0.994–1.106)	0.084		
Gender female	6.771 (0.875–52.38)	0.067	6.091 (0.931–38.5)	0.059
BMI	0.815 (0.675–0.984)	0.033	0.871 (0.770–0.985)	0.028
SLEDAI	0.913 (0.842–0.991)	0.029	0.947 (0.891–1.006)	0.077
CRP			0.979 (0.955–1.004)	0.100
C4	0.926 (0.846–1.014)	0.096		

## Data Availability

No new data were created or analyzed in this study. Data sharing is not applicable to this article.
